# Central nervous system relapse of primary cutaneous anaplastic large cell lymphoma: A case report

**DOI:** 10.1002/jha2.1082

**Published:** 2025-03-06

**Authors:** Satoshi Mitsuyuki, Sayaka Okazaki, Satoru Mukai, Ai Matsuura, Yumiko Yasuhara, Aya Tanaka, Koichi Oshima, Kazuo Hatanaka

**Affiliations:** ^1^ Department of Hematology Sakai City Medical Center Sakai Japan; ^2^ Department of Pathology and Laboratory Sakai City Medical Center Sakai Japan; ^3^ Department of Dermatology Sakai City Medical Center Sakai Japan; ^4^ Department of Pathology Kurume University School of Medicine Kurume Japan

**Keywords:** case report, peripheral T‐cell lymphoma, primary cutaneous anaplastic large cell lymphoma, secondary CNS lymphoma

## Abstract

Primary cutaneous anaplastic large cell lymphoma (PC‐ALCL) has a high relapse rate. However, it typically remains confined to the skin and has a favorable long‐term prognosis. We describe a case of PC‐ALCL that experienced a relapse in the central nervous system (CNS). The patient presented with somatosensory abnormalities in the extremities after local treatment of skin lesions and was diagnosed with CNS relapse of PC‐ALCL. Methotrexate, procarbazine, and vincristine therapy, and alternating brentuximab vedotin, followed by autologous hematopoietic stem cell transplantation (ASCT) cured the CNS lesions, whereas the skin lesions relapsed early. PC‐ALCL could relapse in the CNS; systemic chemotherapy and ASCT may be effective.

## 1 INTRODUCTION

Primary cutaneous anaplastic large cell lymphoma (PC‐ALCL) is a rare type of peripheral T‐cell lymphoma (PTCL) that originates in the skin [1]. Standard treatments include surgical excision or radiation for solitary lesions, while systemic chemotherapy is considered for multifocal lesions. Although the relapse rate is high, the long‐term prognosis is generally favorable because lesions are usually confined to the skin [2]. Herein, we report a case of PC‐ALCL that relapsed in the central nervous system (CNS) and was treated with systemic chemotherapy and autologous peripheral blood stem cell transplantation (ASCT).

## 2 CASE PRESENTATION

A 63‐year‐old man presented to our hospital with somatosensory abnormalities in the fifth finger of his left hand and left lower limb. Ten months ago, the patient had a dark red dermal nodule in the left costal region (Figure S1A), which was pathologically diagnosed as ALCL (Figure S1B–I). A positron emission tomography/computed tomography scanning, along with a bone marrow examination, did not reveal any other lesions. The patient was diagnosed with PC‐ALCL and underwent local radiation therapy (40 Gy). The PC‐ALCL relapsed on the skin of his left knee 4 months ago, for which the patient underwent surgical excision.

**FIGURE 1:**
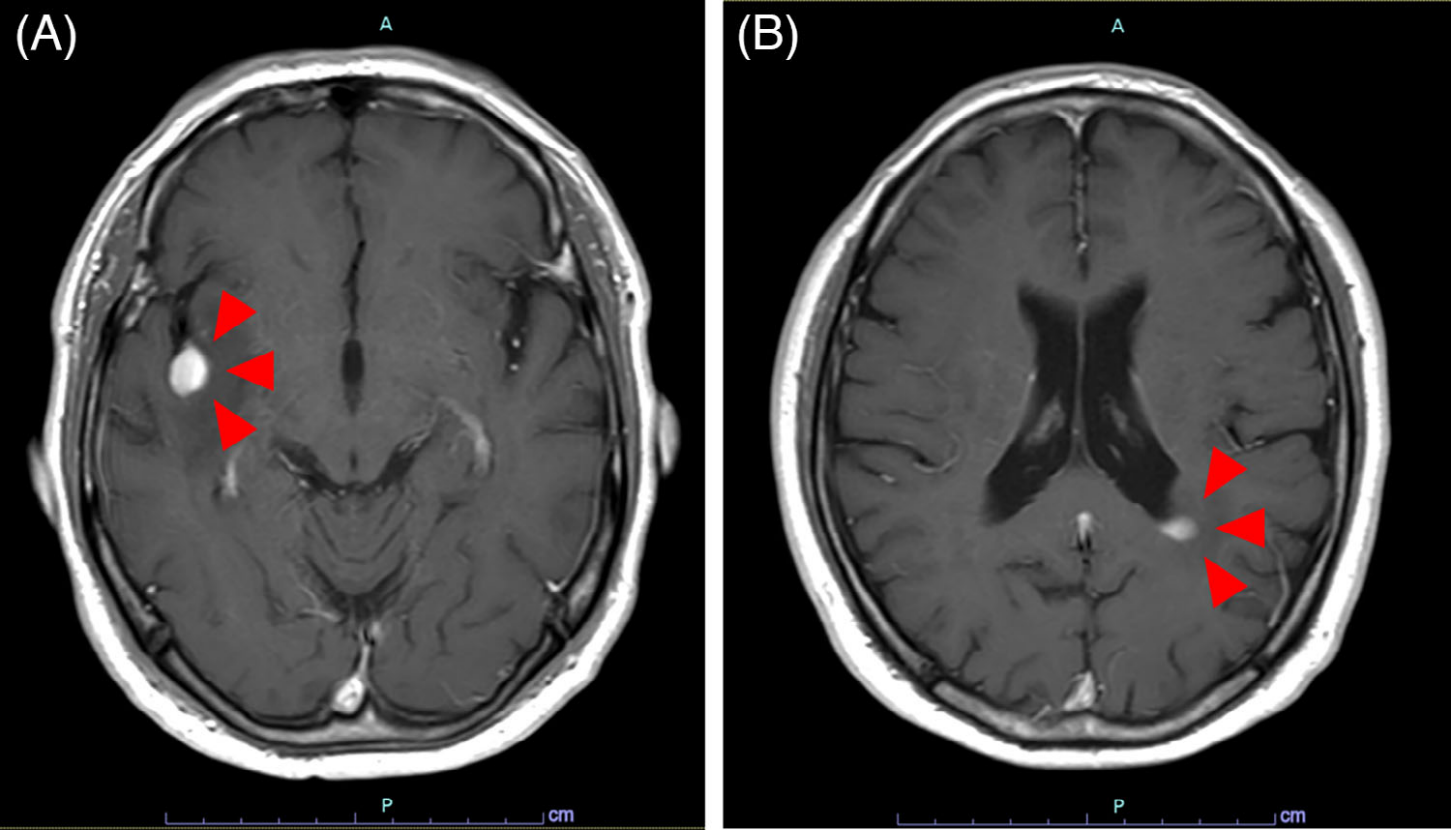
Gadolinium‐enhanced T1‐weighed magnetic resonance imaging shows mass lesions in the right insular lobe (A, red arrowheads) and the left periventricular white matter (B, red arrowheads).

**FIGURE 2:**
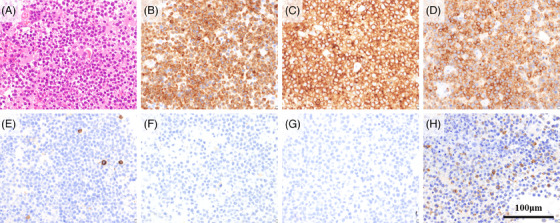
Hematoxylin and eosin staining of the right insular lobe mass biopsy reveals the dense proliferation of large cells with atypical nuclei (A, scale bar: 100 µm). These cells are positive for CD3 (B, scale bar: 100 µm), CD4 (C, scale bar: 100 µm), and CD30 (D, scale bar: 100 µm) on immunohistochemistry staining. They are negative for CD8 (E, scale bar: 100 µm), CD20 (F, scale bar: 100 µm), anaplastic lymphoma kinase (G, scale bar: 100 µm), and epithelial membrane antigen (H, scale bar: 100 µm).

Magnetic resonance imaging (MRI) of the head revealed masses in the right insular lobe and left periventricular white matter (Figure 1A,B). Pathological analysis of a biopsy from the right insular lobe mass showed infiltration of large atypical lymphocytes into the brain tissue (Figure 2A). Immunohistochemically, these atypical lymphocytes were positive for CD3, CD4, and CD30 and negative for CD8, CD20, ALK, and EMA (Figure 2B–H). These findings were consistent with those of the biopsy specimens from the left costal region and left knee. Therefore, a diagnosis of CNS relapse of PC‐ALCL was made. No lymphoma cell infiltration was found in the cerebrospinal fluid. The skin lesions remained in remission without any other systemic lesions. The patient received methotrexate, procarbazine, and vincristine (MPV) chemotherapy, comprising methotrexate (MTX) at a dosage of 3.5 g/m^2^ intravenously on day 1, vincristine at 1.4 mg/m^2^ (capped at 2.0 mg) intravenously on day 1, and procarbazine at 100 mg/m^2^ orally on days 2–8 during odd cycles. Three weeks after starting MPV chemotherapy, the patient was administered brentuximab vedotin (BV; 1.8 mg/kg) intravenously and followed up for 1 week. This treatment schedule was defined as BV‐MPV chemotherapy (one course over 4 weeks) and was repeated three times in total. After the first course, MRI showed partial remission of the CNS lesions. After the second course, an autologous peripheral blood stem cell harvest was performed. Before the transplant, he achieved complete remission. He then underwent ASCT using the BuTT conditioning regimen, which included busulfan (Bu; 3.2 mg/kg) intravenously on days ‐8 to ‐5 and thiotepa (TT; 5 mg/kg) on days ‐4 and ‐3. During the nadir phase, he developed severe febrile neutropenia but this resolved with neutrophil engraftment; the patient was discharged 18 days after the transplantation. Maintenance therapy with BV was planned. However, before the first course, an erythematous papule was seen adjacent to the radiotherapy site (suppinfo1). Furthermore, a similar papule appeared on the left back (Figure 2B). Biopsies of both sites revealed a relapse of PC‐ALCL (Figure S2C–J), occurring 5 weeks after the transplantation. MRI of the head showed no relapse of CNS lesions. He underwent local radiation therapy to the left costal region again (45 Gy) and received salvage therapy with BV, followed by maintenance therapy with dosing every 3 weeks. Two lesions showed a positive response to treatment; both skin and CNS lesions remained in complete remission for 1 year after transplantation.

## 3 DISCUSSION

PC‐ALCL typically manifests only cutaneous lesions or regional lymph node involvement at diagnosis. However, systemic involvement can occur at relapse [3]. Benner et al. reported a 54% recurrence rate, with approximately 10% of patients developing systemic lesions beyond the regional lymph nodes [2]. CNS involvement of PC‐ALCL is even rarer; our literature review identified only three previously reported cases (Table 1) [4, 5, 6]. Cases 1 and 2, similar to the present case, experienced CNS recurrence while their cutaneous lesions remained in remission. As with other lymphomas, the status and progression of systemic lesions may not necessarily correlate with CNS involvement [7].

**TABLE 1:** Summary of reported cases of primary cutaneous anaplastic large cell lymphoma (PC‐ALCL) with central nervous system (CNS) lesion.

Case #	1	2	3	4
Age	59	84	74	63
Sex	Male	Female	Female	Male
Location of cutaneous lesions	Upper and lower limbs	Right upper limb and regional lymph nodes	Scalp contiguous with CNS lesions	Left chest and left knee
Treatment for cutaneous lesions	RT ×7	CHOP ×6	None	RT ×1, Resection ×1
Time from diagnosis to CNS relapse	4 years	6 months	Concurrent	1 year
Systemic or skin lesion at CNS relapse	No	No	Yes	No
Location of CNS lesion	Left frontal lobe	Right temporal lobe	Right frontal and parietal lobes	Right insular lobe and left periventricular white matter
Treatment for CNS relapse	MATRix ×4, ICE ×2, ASCT (conditioning by TT, ETP, CY, CA, and MEL)	None	Dexamethasone, CHOP×1	BV‐MPV×3, ASCT (conditioning using TT and BU)
Outcome	Complete remission 3 years later	Died of cardiorespiratory failure 6 months later	Died of pneumoniae 3 weeks later	Skin lesions relapsed in 1 month, and CNS lesions were in remission 1 year later
Citation	Lebel et al	Simal et al	Seo et al	Our case

Abbreviations: ASCT, autologous hematopoietic stem cell transplantation; BU, busulfan; BV, brentuximab vedotin; CA, cytarabine; CHOP, cyclophosphamide, hydroxydaunorubicin, vincristine, and prednisolone; CNS, central nervous system; CY, cyclophosphamide; ETP, etoposide; ICE, ifosfamide, carboplatin, and etoposide; MATRix, methotrexate, cytarabine, thiotepa, and rituximab; MEL, melphalan; MPV, methotrexate, procarbazine, and vincristine; PC‐ALCL, primary cutaneous anaplastic large cell lymphoma; RT, radiotherapy; TT, thiotepa.

There is no standard treatment for CNS involvement in patients with PTCL, including PC‐ALCL, due to its rarity. Consequently, we referred to treatments used for other aggressive lymphomas. In patients with primary CNS lymphoma (PCNSL) and secondary CNS lymphoma (SCNSL), radiation therapy, intrathecal therapy, and systemic chemotherapy have shown only partial effectiveness [8, 9]. Thus, systemic chemotherapy for induction and high‐dose chemotherapy followed by ASCT for consolidation is recommended in patients with adequate fitness. Omuro et al. reported that induction therapy with R‐MPV chemotherapy, combined with consolidation using high‐dose chemotherapy (thiotepa, busulfan, and cyclophosphamide) followed by ASCT, resulted in favorable disease control for PCNSL, with a 2‐year progression‐free survival rate of 75% and 2‐year overall survival rate of 81% [10]. In the present case, BV‐MPV chemotherapy was used for induction, with rituximab replaced by BV due to CD30 positivity. Regarding consolidation, thiotepa, and busulfan were employed in the high‐dose chemotherapy regimen. Consequently, the skin lesions recurred early. However, remission of the CNS lesions was achieved and maintained for over 1 year. Similarly, Case 1, which employed a high‐dose MTX‐based induction regimen and a thiotepa‐based consolidation regimen, also achieved long‐term remission. There is no data on whether BV could cross the blood‐brain barrier, so BV should be considered as maintenance therapy for systemic lesions because systemic relapse of isolated SCNSL is more likely when CNS lesion‐directed therapy is performed alone [11]. Data on whether BV penetrates the cerebrospinal fluid could provide valuable insights and clarify the rationale behind its use in such treatments.

PC‐ALCL can relapse in the CNS. Although this is a rare but possible event even if skin lesions are in remission. Therefore, careful long‐term follow‐up should be considered. Systemic chemotherapy, including high‐dose MTX and ASCT with a thiotepa‐containing conditioning regimen, may be effective in treating CNS involvement of PC‐ALCL.

## AUTHOR CONTRIBUTIONS

Satoshi Mitsuyuki and Sayaka Okazaki collected clinical data and SM wrote the original manuscript. Kazuo Hatanaka supervised manuscript writing, editing, and reviewing. Yumiko Yasuhara and Koichi Oshima performed histological diagnosis. All authors have read and approved the final manuscript.

## CONFLICT OF INTEREST STATEMENT

The authors declare no conflict of interest.

## FUNDING INFORMATION

This work did not receive any specific grant from funding agencies in the public, commercial, or not‐for‐profit sectors.

## ETHICS STATEMENT

The authors confirm that no ethical approval was required.

## PATIENT CONSENT STATEMENT

Informed consent was obtained from the patient for publication of this case report and any accompanying images.

## CLINICAL TRIAL REGISTRATION

The authors have confirmed clinical trial registration is not needed for this submission.

## Supporting information



Supporting Information

## Data Availability

The data that support the findings of this report are available from the corresponding author upon reasonable request.
