# Supplementary material for: Central nervous system relapse of primary cutaneous anaplastic large cell lymphoma: A case report
Source: EJHaem. 2025 Mar 6;6(2):e1082. doi: 10.1002/jha2.1082 (PMC11883415; doi:10.1002/jha2.1082)
Supplement: Supplementary file 1 — Supporting Information [file JHA2-6-e1082-s001.docx]

**Supplementary materials**

**
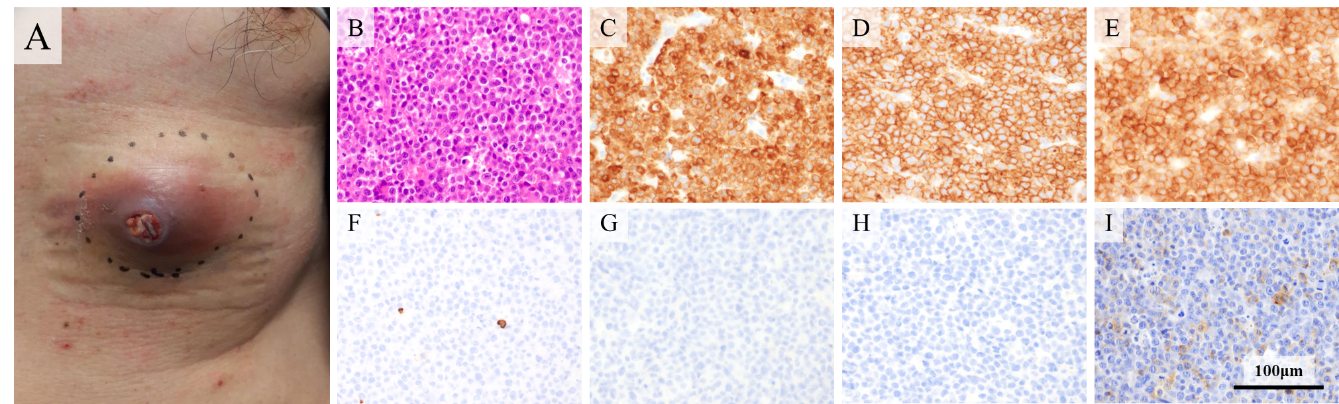
**

**Supplementary Figure 1**: Cutaneous nodular lesion in the left costal region at initial diagnosis.

**
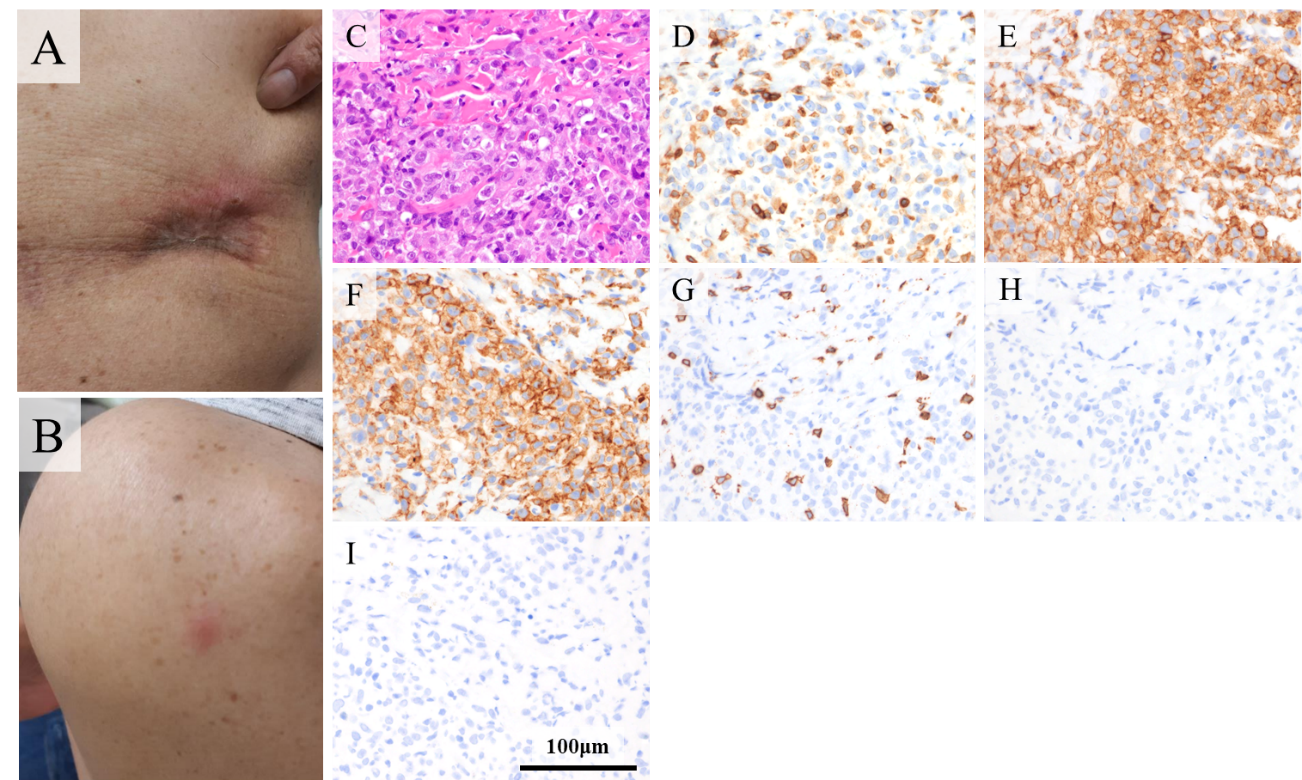
**

**Supplementary Figure 2**: Erythematous papules on the left costal region and left back after transplantation.
